# Nano-Charged Polypropylene Application: Realistic Perspectives for Enhancing Durability

**DOI:** 10.3390/ma10080943

**Published:** 2017-08-14

**Authors:** Carlo Naddeo, Luigi Vertuccio, Giuseppina Barra, Liberata Guadagno

**Affiliations:** Department of Industrial Engineering University of Salerno Via Giovanni Paolo II, 132-84084 Fisciano (SA), Italy; lvertuccio@unisa.it (L.V.); gbarra@unisa.it (G.B.)

**Keywords:** polymer durability, thermoplastic nanocomposites, nanocomposites photo-oxidation, carbon nanotubes

## Abstract

Isotactic polypropylene/multi-walled carbon nanotube (iPP/MWCNTs) films have been exposed to accelerated weathering in a UV device for increasing times. The effect of UV irradiation on the structural and chemical changes has been investigated. The resistance to accelerated photooxidation of (iPP/MWCNTs) films has been compared to the photooxidation behaviour of unfilled polypropylene films with the same structural organization. The chemical and structural modifications resulting from photooxidation have been followed using infrared spectroscopy, calorimetric and diffractometric analysis. MWCNTs embedded in the polymeric matrix are able to strongly contrast the degradation mechanisms and the structural and morphological rearrangements caused by the UV treatment on the unfilled polymer. MWCNTs determine an induction period (IP) before the increase of the carbonyl and hydroxyl groups. The extent of the IP is strictly correlated to the amount of MWCNTs. The low electrical percolation threshold (EPT) and the electrical conductivity of the nanocomposites, together with their excellent thermal and photooxidative stability, make them promising candidates to fulfill many industrial requirements.

## 1. Introduction

The interest in applying nanoscaled fillers into polymeric matrices arises from the possibility to transfer the very interesting and unique properties of nanometric particles to polymeric systems able to be applied for specific functional properties. In this context, nanostructured fillers such as clay, carbon nanotubes (CNT) and graphene-based nanoparticles have aroused great enthusiasm in the scientific community given the possibility to transfer the very interesting nanostructure properties to polymeric matrices. This last strategy has proven to be very effective to impart or improve electrical, thermal, sensing, mechanical, transport properties of polymeric systems [1,2,3,4,5,6,7,8,9,10,11,12]. Unlike traditional filled polymer systems, nanocomposites require a very low dispersant loading (between 0.01 wt % and 3 wt %) to achieve significant enhancements in physical and chemical properties. As result of these enhancements, nanocomposites are expected to play a significant role in future industrial applications. The prospect of producing materials with tailored physical and electronic properties could result in interesting industrial applications ranging from aeronautics, to photooxidation or corrosion prevention to electronic/automotive parts to industrial equipment and several others. The usefulness of many polymeric nanocomposites designed for outdoor applications strongly depends on their durability in environments in which they are employed. The degradation of polymers entails several physical and chemical processes involving structural and morphological changes [13,14,15,16,17], which cause a significant decrease in the mechanical performance and as result the reduction of the service life.

Thus, the study of degradation of polymer nanocomposites is an extremely important area from a scientific and industrial point of view and a better understanding of degradation mechanisms can greatly contribute to extend the material durability or to open other application fields, which require durability performance not currently available among commercial polymeric materials. In addition to the processing parameters, it is well known that nanocomposite properties strongly depend on the nature of nanofillers, polymeric matrix, different additives and processes of preparation.

Conventional additives (stabilizers/sensitizers) are typically used for specific purposes. As an instance, the stabilizers-based hindered phenols are for reducing/retarding the oxidation processes by donating hydrogen to the radical [18]. On the contrary, the additives based on nanoparticles are generally used for improving thermal, physical, optical and electrical properties of the polymers [18].

Nanoparticles, such as organically modified montmorillonite (OMMT) nanofillers, can have negative impact on the durability of the nanocomposite materials under UV-light exposure. Photo-oxidation of iPP filled with montmorillonite (MMT) has been extensively studied [19,20,21,22,23,24] and it is established that iPP nanocomposites unfortunately degrade faster than the pristine polymer. It has been hypothesized that this is due to the degradation of alkyl-ammonium cation exchanged in MMT and the catalytic effect by nanofiller impurities that could catalyze the decomposition of hydroperoxide formed by matrix photo-oxidation [25,26,27,28]. The effect of some other nanofillers, such as CNTs, or graphene-based nanoparticles can be tuned. Among the nanoparticles commercially available, carbon nanostructured forms, embedded in thermoplastic and thermosetting polymeric systems, have proven to be very effective for slowing down the photooxidation reactions. Multi wall carbon nanotubes (MWCNTs) embedded in syndiotactic Polypropylene (sPP) films, crystallized in more usual Form I, contribute to strongly decrease the rate of photooxidation reactions simultaneously increasing oxidative thermal stability of the polymeric matrix [29,30]. The ability of MWCNTs to interact with oxygen molecules making them unavailable in the first stages of photooxidation reactions has been hypothesized to be responsible for a such beneficial behavior. The advantage of CNTs for enhancing the photo-stability of polymeric matrix has also been found for different polymeric systems. Dintcheva et al. studied the influence of unfunctionalized MWCNTs and MWCNTs functionalized with –COOH groups on the photo-stability of Polystyrene-Polybutadiene-Polystyrene (SBS) [31]. Compared with the photo-oxidation of unfilled SBS, the nanocomposites showed a significant improvement in the photo-stability, due to the presence of MWCNTs and even more, of MWCNTs-COOH, which were found able to efficiently protect SBS against UVB exposure. This interesting influence of MWCNTs has been attributed to their acceptor-like electron properties and their ability to shield UV-light. Besides, this nanofiller can act as radical traps, hindering the crosslinking and slowing down the oxidation process [31].

The influence of carbon nanotubes was also studied for the Poly(ethylene-co-vinyl acetate) (EVA) photodegradation [32]. CNTs were found to act as inner filters and antioxidants, which contribute to reduction in the rate of photooxidation of the polymeric matrix. Concentration, morphology and functionalization of CNTs were found to affect the global rate of photooxidation of the polymeric matrix [32]. Dintcheva et al. studied the resistance to accelerated photo-oxidation of polyethylene/multi-walled carbon nanotubes (MW-CNTs) composite films. The behavior of the nanocomposites was compared with that of the unfilled polyethylene films. At short exposure time, i.e. less than 200 h, the rates of carbonyl formation were found very similar to that observed for unfilled polyethylene film but at longer irradiation times the carbonyl formation was found to increase for lower MW-CNTs contents (0.1 wt %, 0.2 wt % and 0.5 wt %), and to decrease for higher MW-CNTs contents (1 wt % and 2 wt %) [33]. The stabilization effect of MWCNTs was also found for other polymers: epoxy [34,35,36,37], Thermoplastic Polyurethane (TPU) [38], Polyamide 6,6 (PA6) [39,40,41], PLA [42] and isotactic polypropylene (iPP) [40,43]. Epoxy containing MWCNTs exposed to UV radiation degraded at a much slower rate than the unfilled epoxy or the epoxy/nanosilica composite [37]. The behaviour of nanocomposites of PE and iPP containing embedded MWCNTs (3 wt %) has been studied by Bocchini et al. through FT/IR investigation [43]. This behaviour was found to be dependent on the type of polymer and on the presence or absence of antioxidant. In that paper, it was also found that the inclusion of MWCNTs affected the photooxidation of the two polymers working as an UV-screener, on the other hand the increase of thermal oxidation provoked by conversion of photon energy into thermal partially balance the decrease of positive effects on oxidation rate. The author concluded that negative degradation effects could be minimized by the presence of antioxidants that show interesting synergistic effects with MWCNTs.

Despite the large industrial application of the monoclinic polymorphic form (α form) of isotactic polypropylene (iPP) and the well-known improvement of several properties of its nanocomposites based on the inclusion of MWCNTs [44,45,46,47,48], few efforts have been made on the effect of MWCNTs on the photo-oxidative behavior of nanocomposites based on iPP Polymer. Isotactic Polypropylene is the lightest polymer and the α polymorph is employed in a wide range of applications such as fiber, packaging, the automotive industry, construction materials and nondurable goods. The excellent mechanical properties (toughness), resistance to chemicals, processability and low cost of iPP make it a more promising candidate for several outdoor applications. iPP is the most widely used thermoplastic since it is very cheap and flexible for molding. However, its application, without stabilizers, is limited due to its relatively poor photooxidation stability.

This study is aimed at evaluating the photo-oxidative behavior of thermoplastic nanocomposites, based on isotactic Polypropylene (iPP) without the presence of stabilizing antioxidants where the crystalline phase is in the more common α monoclinic form, whereas the amorphous phase contains embedded different percentage of MWCNTs. The performed work highlights that the nanofilled polymer shows high stability to the photo-oxidative degradation and hence it can also be applied for its electrical conductivity, one of the most relevant functional property imparted by MWCNTs to the Polymer (see section “Electrical Properties”).

This work arises from the need to better understand if the relevant functional properties imparted by MWCNTs to the iPP polymer can be simultaneously coupled with the desired photooxidative stability. The results here discussed highlight that the formulated nanocomposites are promising candidates for outdoor applications due to their impressive photooxidative stability which allows to fully exploit functional properties of nanocomposites loaded with conductive nanoparticles.

Another issue related to the use of CNTs/composites concerns the risk assessment; it is of relevant importance to know whether CNTs from nanocomposites can be released into the environment or if they remain embedded in the matrix. This topic has been deeply investigated by Schlagenhauf et al. [49] and Hirth at al. [50]. It has been concluded that, in the case of ductile matrix materials, the ductile matrix is able to reflow around the CNTs during fragmentation [38] and no or only few protruding CNTs from abraded particles are expected.

## 2. Experimental Section

### 2.1. Materials and Preparation of Samples

Unstabilized Isotactic polypropylene (iPP) characterized by high flow resin for easy moldability, excellent dimensional stability and low built-in stress were obtained in form of pellets from Aldrich Polymer Products (Milan, Italy). The properties of the Polymer are listed in Table 1.

MWCNTs (3100 Grade) were from Nanocyl S.A. (Sambreville, Belgium). They are characterized by an outer diameter ranging from 10 to 30 nm and a length from hundreds of nanometres to some micrometre. Walls number varies from 4 to 20 and the specific surface area is around 250–300 m^2^/g (Brunauer–Emmett–Teller method) [51]. The carbon purity is higher than 95 wt % and the metal oxide impurity is lower than 5 wt % (thermogravimetric analysis). A reference iPP sample and iPP/MWCNTs composite samples were prepared using a laboratory-scale conical twin-screw extruder (ThermoHakke Micro Compouder Minilab Rotor, USA). Polymer matrix and carbon nanotubes were premixed manually and then loaded into the compounder. The premixing process has been performed using the following procedure: iPP pellets were molded in a hot press (Carver Inc., Wabash, IN, USA), at 180 °C, forming 70 ± 5 µm thick films. Subsequently, the films were used to prepare sandwiches containing in the middle the exact amount of CNTs. These films, in the form sandwich, were molded in the hot press again and finally shredded (in the form of thin, small squares) before the extrusion process.

All batches, weighting 6 g each, were processed at 170 °C with a screw speed of 200 rpm for 20 min. Upon completion of blending, the molten polymer was ejected from the extrusion and allowed to cool at ambient temperature. The extruded samples were moulded in a hot-press (Carver Inc.) at 180 °C forming 75 ± 5 µm thick films which were rapidly quenched in a bath with water at 100 °C. The quenching at 100 °C has been chosen because it guarantees the formation of semicrystalline film where the crystalline phase is in the more usual α form. Furthermore, this experimental procedure has allowed a strong reproducibility in the phase composition of the unfilled and filled iPP films. The samples are coded as AX t were A is isotactic polypropylene, X is the weight percentage of MWCNTs and t is the ultra-violet irradiation time in h.

### 2.2. Characterization Methods

Differential Scanning Calorimetry (DSC) was carried out on unirratiated and photo-oxidated iPP/MWCNT samples (10 ± 0.5 mg) using a thermal analyzer Mettler DSC 822/400 (Mettler-Toledo Columbus, OH, USA) equipped with DSC cell purged with nitrogen and chilled with liquid nitrogen for sub-ambient measurements. Temperature range was 0–300 °C at a heating rate of 10 °C/min. Calorimetric traces were utilized for evaluate the melting point and crystalline modifications of analyzed samples. Thermogravimetric analysis (TGA) was carried out in air and nitrogen on unirratiated and photo-oxidated iPP/MWCNT samples (5 ± 0.5 mg) using a Mettler TGA/SDTA 851 thermal analyzer (Mettler-Toledo AG Schwerzwnbach CH). Temperature range was 25–900 °C at a heating rate of 10 °C/min. Thermogravimetric traces were analyzed for evaluating the weight loss and thermodegradation temperature at weight loss of 5 wt % (*T*_d_) for all samples. All DSC and TGA measurements were repeated at least three times. X-ray diffraction (XRD) were performed with a Bruker D8 Advance diffractometer (Bruker Axs Inc., Madison, WI, USA) with Ni-filtered Cu_Ka_ radiation (λ = 1.54050 Å). From the diffractometric profiles of the analyzed samples is detected the size of crystallites D_hkl_ in the direction perpendicular to the reflecting (hkl) planes using the Scherrer equation D_hkl_ = Kλ/ωcos θ where λ is the wavelength, K is a costant value of 0.9, ω is width at half height of the peak and θ is half of the diffraction angle.

In order to study the growth of carbonyl (CO) and hydroxyl (OH) bands during UV photodegradation of samples, FTIR measurements were performed by using a Bruker Vertex 70 FT-IR spectrophotometer (Bruker Optics Inc., Billerica, MA, USA) in absorbance mode with a resolution of 4 cm^−1^ (32 scan collected) between 400 and 4000 cm^−1^. For the polyolefins, the peak absorbance at 1713 cm^−1^ was assigned to carboxylic acids forming and the peak absorbance at 3340 cm^−1^ was assigned to hydroxyl moieties. The carbonyl and hydroxyl concentrations are calculated respectively by the relations [CO] = DO_co_/ε_1713_ t ρ_pp_ and [OH] = DO_OH_/ε_3340_ t ρ_pp_; where [CO]_av_ and [OH]_av_ are the average carbonyl and hydroxyl concentrations expressed in mol kg^−1^, DO_co_ is the 1713 cm^−1^ peak absorbance value, DO_OH_ is the 3340 cm^−1^ peak absorbance value, t is the sample thickness (in cm), ρ_pp_ is the polypropylene density (0.90 kg L^−1^), while ε_1713_ = 300 L mol^−1^ cm^−1^ and ε_3340_ = 70 L mol^−1^ cm^−1^ are the molar absorptivity [52,53]. The bands where there is overlapping were decomposed into their different components using a complex fitting in which a Lorentzian and Gaussian contribution were considered in the form:

(1) $$f(x)=(1-L)H\;\exp-[{\left(\frac{x-x_{0}}{w}\right)}^{2}(4\ln2)]+L\frac{H}{4{\left(\frac{x-x_{0}}{w}\right)}^{2}+1},$$

where *x*_0_ is the peak position, *H* is the height, *w* is the width at half-height and *L* is the Lorentzian component.

### 2.3. Accelerated Weathering Treatment

The photooxidative degradation was carried out by exposing the samples (75 ± 5 µm thick film) to an accelerated weathering tester (QUV/Spray Q-Panel Lab Product, Westlake, OH, USA) at 45 °C with solar eye irradiance control system and fluorescent UV-A lamps (UVA 340). Lamps of this kind have no ultraviolet output below the normal solar cut-off of 295 nm. They are the best available simulation of sunlight in the critical short wavelength UV region between 365 nm and the solar cut-off of 295 nm. The emission peak is at 340 nm with an irradiance level of 0.78 W/m^2^. The radiative treatment was performed at the temperature of 45 °C in the absence of water spray.

### 2.4. Electrical Properties

The volume electrical resistivity was carried out according to Cabot Test Method (CTM) E043 based on ASTM D4496. Two silver paint electrodes located at the sample edges were used. The specimen length was 30 mm with 5 mm long electrodes, leaving an effective span (*L*) of 10 mm between the silver electrodes, the specimen width was 10 mm and its nominal thickness was 75 ± 5 µm. In order to minimize surface effects in the measurements, silver paint electrodes were painted completely covering the ends of the specimens. A DC voltage was applied between the electrodes and the volume electrical conductivity (σ) was calculated using the measured electrical resistance (*R*) and the specimen dimensions as

σ = *L*/*AR*, (2)

where *A* is the cross-sectional area of the specimen.

## 3. Results and Discussion

### 3.1. Thermogravimetric Results

**Figure 1:**
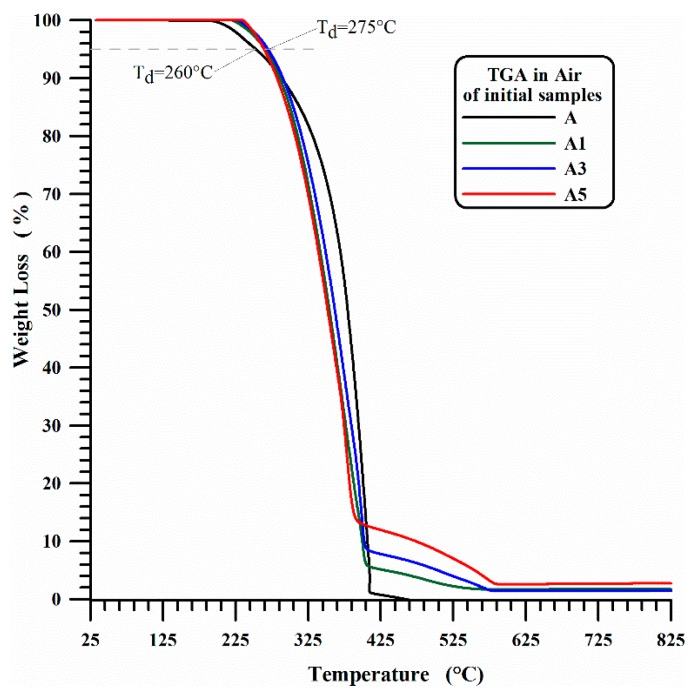
TGA in air of initial iPP (A) and iPP-MWCNT nanocomposites (A1, A3, A5).

**Figure 2:**
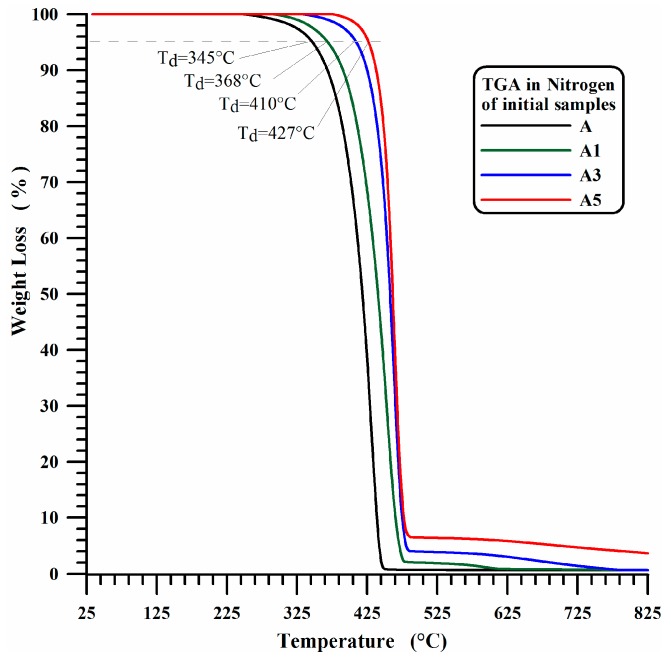
TGA in nitrogen of initial iPP (A) and iPP-MWCNT nanocomposites (A1, A3, A5).

Unfilled iPP films (A1) and nanocomposites filled with 1 wt % of MWCNTs (A1), 3 wt % of MWCNTs (A3) and 5 wt % of MWCNTs (A5) have been analyzed by means of thermogravimetric analysis before and after the UV-treatment. The thermogravimetric curves in air and nitrogen of the unirradiated unfilled iPP (A) and the filled A1, A3 and A5 samples are shown in Figure 1 and Figure 2. The presence of MWCNTs in A1, A3 and A5 films increases the thermodegradation temperature (*T*_d_) of 15 °C for thermogravimetric measurements in presence of air.

A more marked increase, which is strongly dependent on the percentage of MWCNTs, is detected for measurements carried out in presence of nitrogen. In particular, increases in *T*_d_ of 23, 65, and 82 °C are observed with increasing MWCNT of 1 wt %, 3 wt % and 5 wt % respectively.

**Figure 3:**
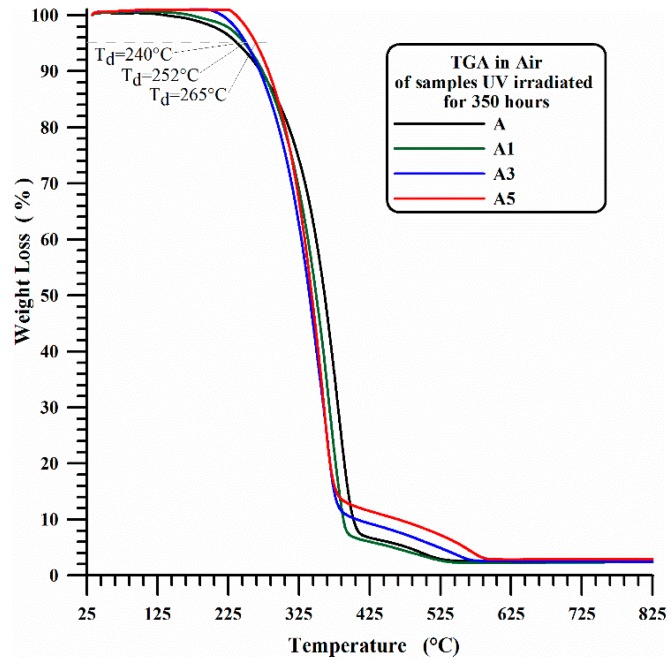
TGA in air of iPP (A) and iPP-MWCNT nanocomposites (A1, A3, A5) after 350 h UV irradiation.

**Figure 4:**
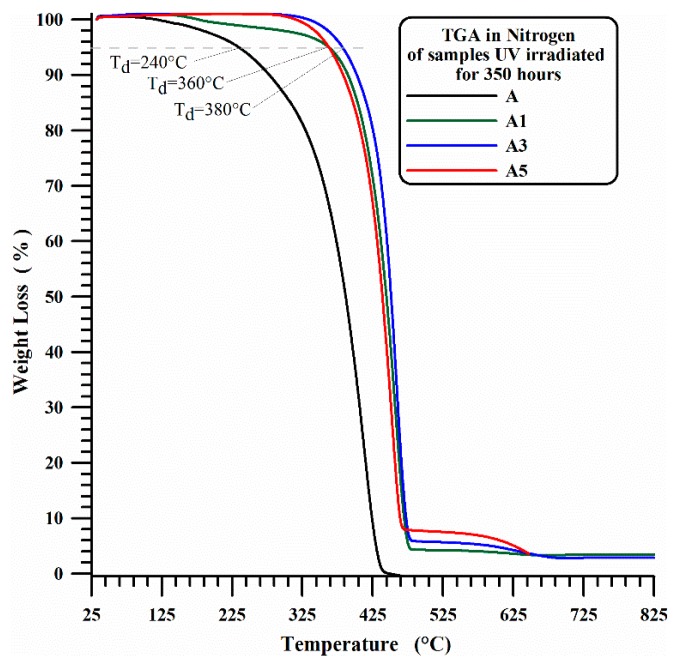
TGA in nitrogen of iPP (A) and iPP-MWCNT nanocomposites (A1, A3, A5) after 350 h UV irradiation.

In this last case, as expected, the beginning of the first stage of thermal degradation is most probably due to degradation processes which do not involve oxygen (dehydration, random scission etc.), which are activated at higher temperature with respect to the degradation mechanisms in presence of O_2_ molecules (see Figure 1). It is worth noting that the presence of MWCNTs slows down the beginning of the thermal degradation of temperature ranges that seem to be directly related to the amount of nanofiller. Figure 3 and Figure 4 show the thermogravimetric curves in air and nitrogen carried out after 350 h UV-irradiation for A, A1, A3 and A5 samples. In air, the beginning of the thermal degradation (*T*_d_) for irradiated samples is 240 °C for unfilled iPP (A), 252 °C for iPP with 1 wt % and 3 wt % of MWCNT (A1, A3) and 265 °C for iPP with 5 wt % of MWCNT (A5). In inert ambient (nitrogen), the thermodegradation temperature (*T*_d_) increases from 240 °C for neat iPP (A) to 360 °C for iPP with 1 wt % and 3 wt % of MWCNT (A1, A3) and to 380 °C for iPP with 5 wt % of MWCNT (A5).

**Figure 5:**
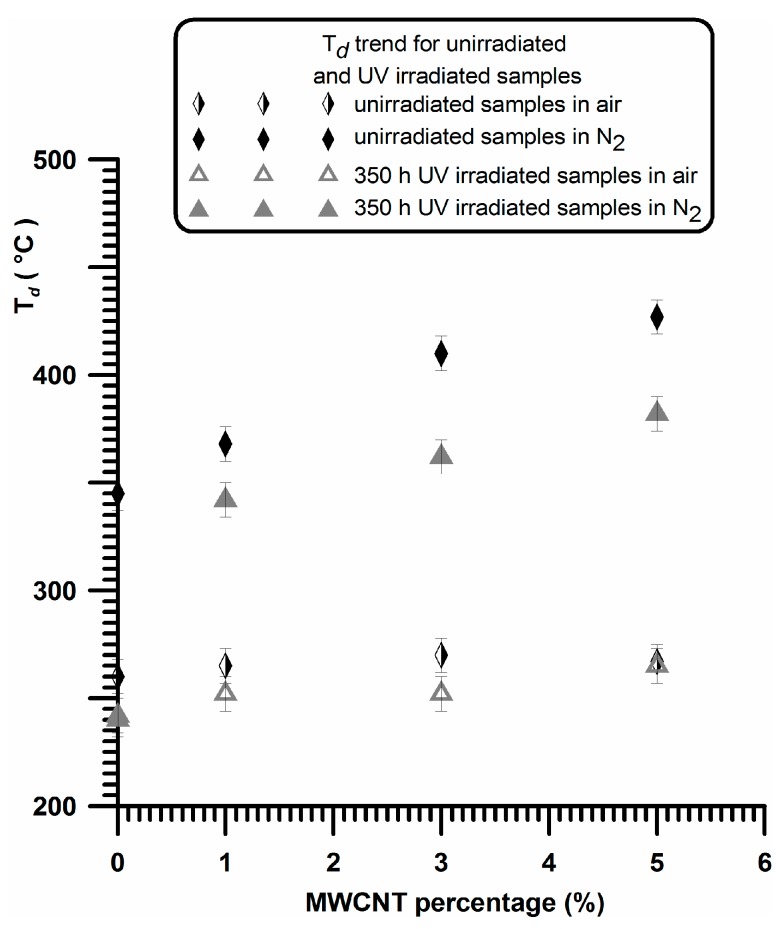
*T*_d_ as function of MWCNT percentage for unirradiated and 350 h UV irradiated samples.

The overall trend of *T*_d_ as function of MWCNT percentage for unirradiated and 350 h UV irradiated samples is shown in Figure 5.

For sample A, the UV irradiation produces a slight reduction of thermodegradation temperature *T*_d_ (from 260 °C to 240 °C) in air and a more evident reduction of T_d_ (from 345 °C to 240 °C) under a flowing of nitrogen atmospheres, confirming what is usually reported in the current literature. In fact, in Ref. [54] the authors performed TGA analysis of polypropylene in air atmosphere, and they found that the TGA traces shifted to lower temperatures with increasing weathering time, and a similar trend was also found in literature for TGA analysis, performed under Nitrogen atmosphere, on commercial polypropylenes before and after 250 h [55] or 300 h [56] of exposure to the UV irradiation.

The results from thermogravimetric experiments highlight that, as already stated in Ref. [57], the MWCNTs produce a protection action type a barrier effect, which prevents the transport of the products of photodegradation of polymer matrix from the solid (condensed) to the gaseous phase. This protection action may be due to the restriction of mobility of macromolecules caused by the presence of MWCNTs. As the restriction sites increases, there is a reduction in tension of C–C bond induced by thermal action and hence the *T*_d_ increases.

### 3.2. DSC Experiments

**Figure 6:**
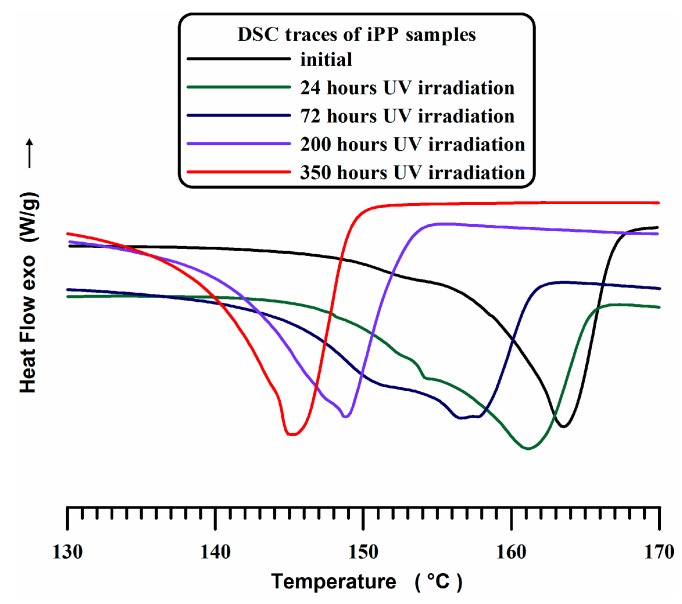
DSC traces of pure iPP (A) sample subject to UV irradiation at increasing times.

DSC traces of pure iPP (A) sample subject to UV irradiation at increasing times are shown in Figure 6.

The endothermic peak, centered between 162 °C and 167 °C, corresponds to the usual melting temperature of the iPP α polymorph. The profile of the peak, characterized by a significant broadening on the left side and a wide weak endotherm between 150 °C and 158 °C, which precedes the main endothermic peak, is due to melting-recrystallization-melting mechanism [58,59]. This phenomenon is well known in literature, it is typical of semicrystalline polymers; the perfecting of the less stable original lamellar crystallites is assumed to take place during heating scan leading to the appearance of a second higher melting endotherm, which represents the melting of the recrystallized crystals [60,61]. The irradiation treatment strongly influences the profiles of the DSC curves. In fact, the main endothermic peak progressively shifts at lower temperatures as the degradation goes on. For an irradiation time of 350 h, a relevant decrease of about 20 °C is observed. This is clear evidence that the degradation phenomenon is at an advanced stage. In fact, the scission of chains, on the crystallite boundary surfaces, causes a reduction of their dimensions determining the lowest value of the melting temperature observed in the calorimetric trace of the iPP irradiated 350 h.

**Figure 7:**
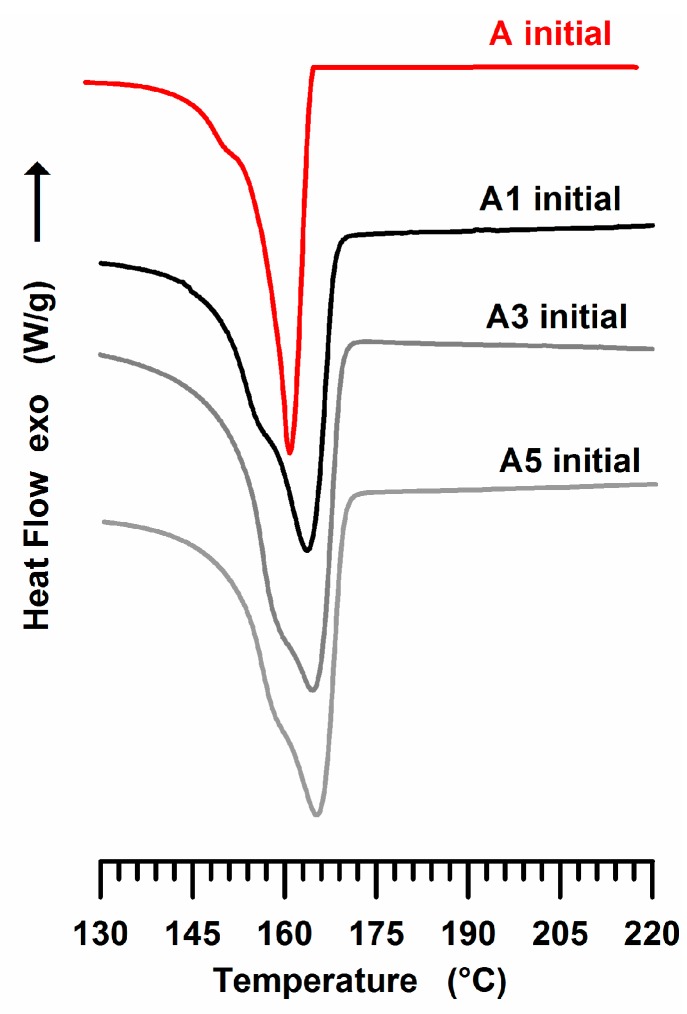
DSC traces of iPP (A) sample and iPP-MWCNT nanocomposites (A, A1, A3, A5).

Figure 7 shows the thermograms of the unirradiated samples A1, A3 and A5 together with that of the unfilled sample (A) for comparison. The wide weak endotherm between 150 °C and 158 °C in the initial unfilled sample shifts at higher temperature in the nanofilled samples, determining the prominent shoulder on the left side of the endotherm and hence its double-peak profile.

**Figure 8:**
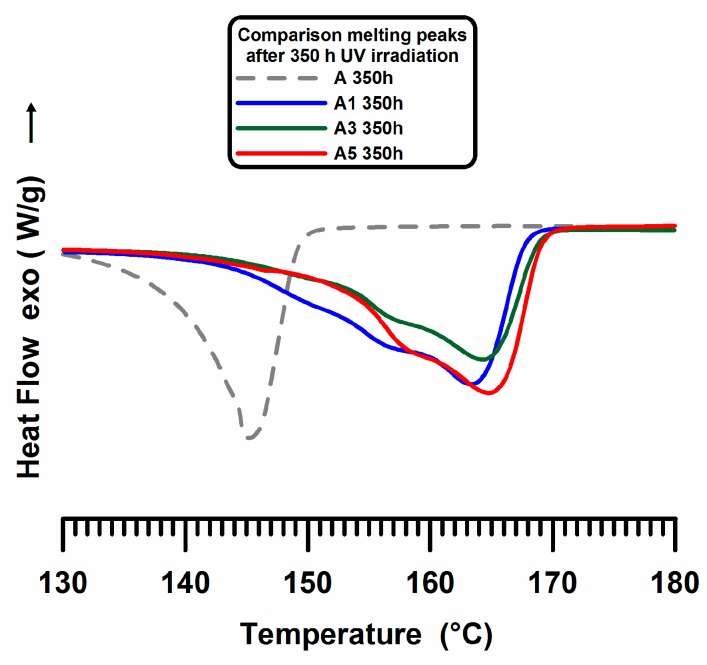
Calorimetric-melting endotherm traces of A, A1, A3 and A5 samples after 350 h of UV irradiation.

This behaviour can be due to the effect of nanofiller; the carbon nanotubes, acting as nucleating agents for iPP crystals, determine an increase of the lamellar thickness of less stable original crystallites. This effect has been found also for other fillers; iPP loaded with inorganic fillers (Talc) shows a very similar behavior [58]. Figure 8 shows the thermograms of iPP samples containing dispersed 1 wt % and 3 wt % and 5 wt % of MWCNT (A1, A3 and A5) exposed to the UV irradiation time of 350 h. The DSC curve of the unfilled sample (A) is also shown for comparison (see dashed black curve).

It is worth noting that the presence of MWCNTs, also for A1 sample, which corresponds to the lowest amount of nanofiller (see blue trace), greatly slows down the breaking of the polymeric chains.

**Figure 9:**
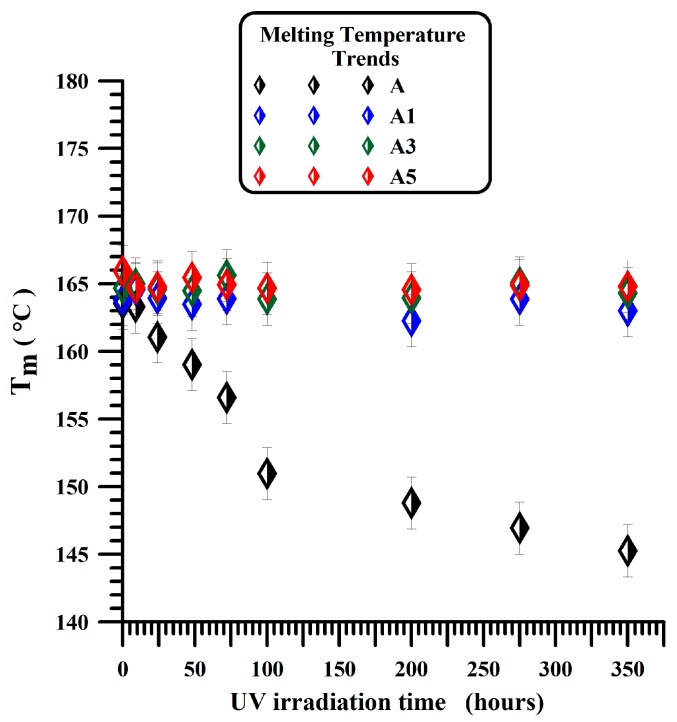
Melting temperature vs UV irradiation times of iPP sample (A) and photooxidated nanocomposites (A1, A3, A5).

The described trend is shown in Figure 9. For the samples containing embedded MWCNTs (A1, A3 and A5 samples), no substantial changes can be detected in the investigate range of time. All points of A3 and A5 samples fall in the same straight line and a similar behavior is detected for A1 sample.

**Figure 10:**
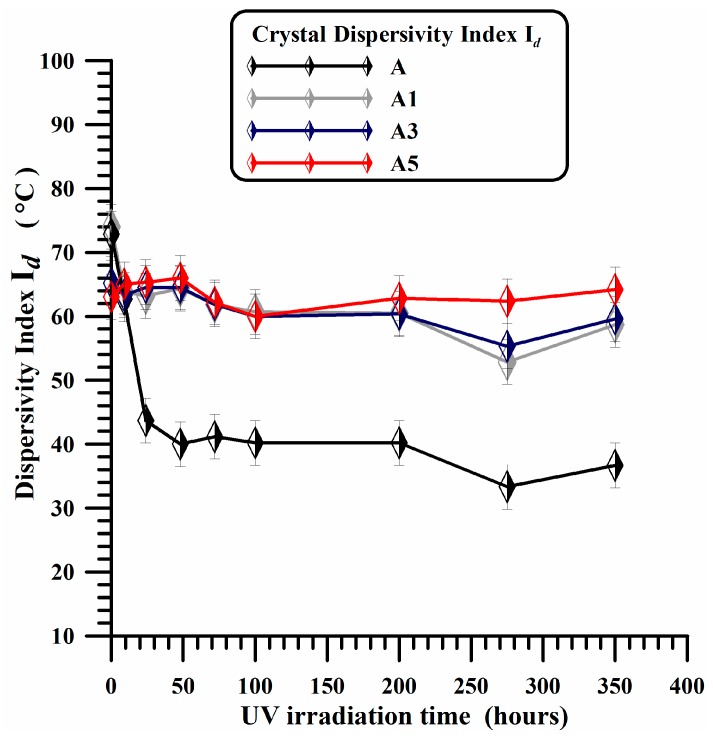
Dispersivity index vs UV irradiation times of A, A1, A3 and A5 samples.

As discussed before, for A1, A3 and A5 samples, only small changes in the profile of the main endothermic peak are observed with respect to the unfilled sample. The different profile of the melting peak suggests a different morphological arrangement in the distribution of the crystal thickness. The dispersity of crystal thickness in each sample can be represented by *I*_d_ = *T*_m_ − *T*_0_, where *T*_m_ is the peak melting temperature and *T*_0_ the onset temperature, corresponding to the melting of crystallites with smaller sizes [62]. The onset temperature is determined from the intersection of the slope on the left of the melting endotherm and the baseline. The values of Id as a function of the time are shown in Figure 10 for all analyzed samples. For the unfilled sample (A), a strong and rapid decrease is observed in I_d_ value in the first 25 h of irradiation time; afterward an almost constant value is observed up to 200 h, before a further slight decrease for longer irradiation times. A very weak decrease in the first 25 h of irradiation treatment is observed instead in the crystal dispersity index of the A1 sample, whereas for A3 and A5 samples, no appreciable changes are observed in the trend of Id up to 200 h. It is worth noting that, for A5 sample, the experimental points indicate a nearly constant trend for all investigated times. This is a clear evidence that, on the contrary of what happens for the unfilled sample, carbon nanotubes embedded in the polymeric matrix are able to strongly contrast the degradation mechanisms and hence the structural and morphological rearrangements due to the UV exposure.

The dispersity of crystal thickness (*I*_d_) in the initial nanofilled samples is slightly smaller than the initial *I*_d_ of the unfilled sample; it remains almost constant in the investigated range of irradiation time. In conclusion, this result highlights the high photooxidative stability of the samples containing MWCNTs.

### 3.3. Wide-Angle X-ray Scattering

**Figure 11:**
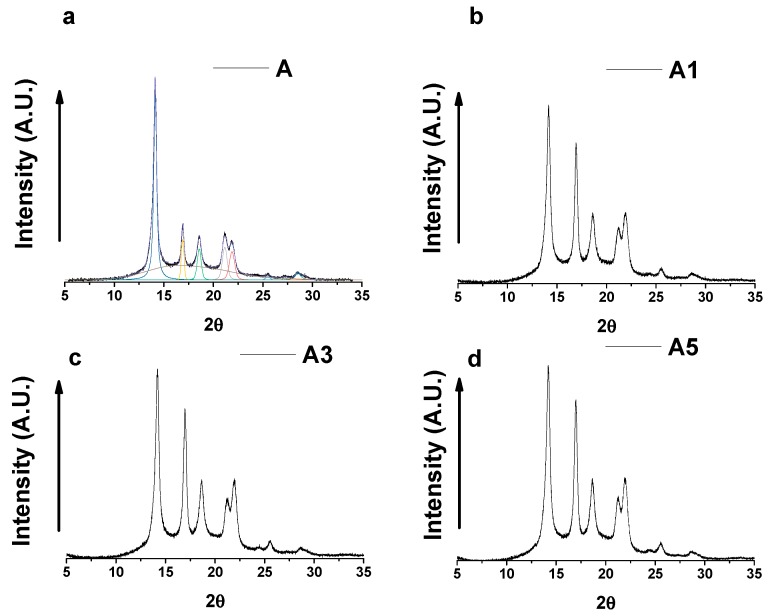
X-ray diffractograms of samples A, A1, A3 and A5.

For better understanding the structural rearrangements determined by the photooxidative reactions, X-ray diffractometric analysis has been carried out for unfilled and filled samples. X-ray diffractograms of the samples are shown in Figure 11. In the diffractogram of sample A, we observe the peaks of the most common crystal form in iPP that is the α-phase having a monocline crystal structure. In fact, the peaks characteristic of the α phase at 2θ = 14.1°, 2θ = 17.0°, 2θ = 18.6°, 2θ = 21.3°, 2θ = 21.9°, 2θ = 25.4° and 2θ = 27.2° corresponding to the (110), (040), (130), (111), (131)/(041), (150)/(060), (200) reflections are observed. From the diffractogram profiles, it is possible to derive the crystallinity of each sample as the ratio of the integral intensity under all crystalline peaks (sum of integral intensities under the crystalline peaks) and amorphous halo. The results show a slight decrease in crystallinity degree from 57 wt % for iPP to 54 wt %, 52 wt %, 51 wt % for A1, A3, A5 respectively. The crystallinity degree tends to slightly decrease with increasing the MWCNTs amount.

It is well known that crystallites smaller than 120 nm create broadening of diffraction peaks, this peak broadening can be used to quantify the average crystallite size of nanoparticles using the Scherrer equation. Crystallite size is a measure of the size of a coherently diffracting domain.

Due to the presence of polycrystalline diffracting domain aggregates, crystallite size may not be the same thing as particle size. Paul Scherrer first observed that small crystallite size could give rise to peak broadening. He derived a well-known equation for relating the crystallite size to the peak width. As described in the experimental section, from the diffractometric profiles, the crystallite dimensions, using the Scherrer equation, have been evaluated. The half-height width of the reflections (β) has been calculated after the deconvolution of the X-ray profiles, as shown in the X-ray diffractogram of sample A.

**Figure 12:**
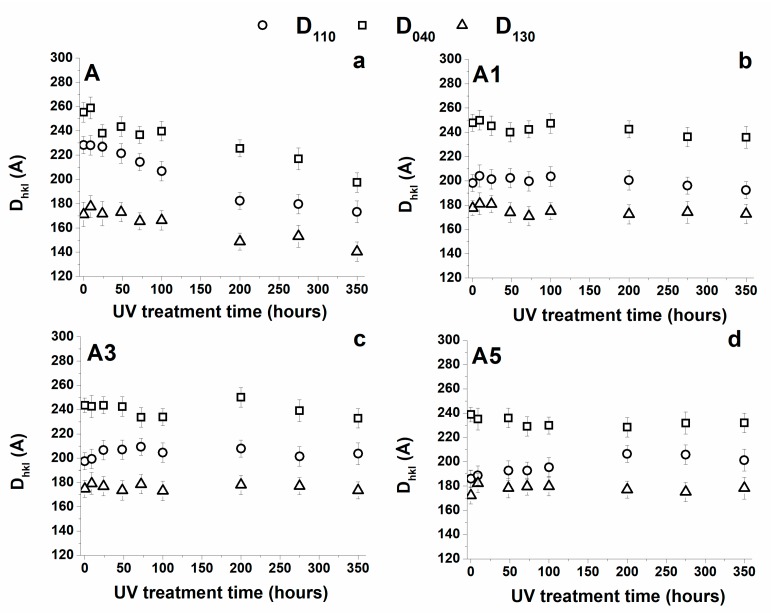
Trend of the Crystallite Coherence Lengths Perpendicular to Reflection Planes 110 (D110), 040 (D040), 130 (D130) for the unfilled and filled samples as a function of UV treatment time.

The Crystallite Coherence Lengths Perpendicular to Reflection Planes 110 (D110), 040 (D040), 130 (D130) for the unfilled and filled samples as a function of UV treatment time are shown in Figure 12.

For the unfilled sample (A), the UV treatment tends to reduce the size of the crystals. The lower values are observed in the samples treated for the maximum investigated time. This result can be explained considering that, for extended times of exposure, the progression of the photooxidative phenomena has reached an advanced stage. Most probably, the degradation is extended up to the crystallites surfaces conditioning their dimensional stability. In fact, a scission of chains, on the crystallite boundary surfaces, disturbing the order of molecular segments adjacent to the crystals determines a reduction of their dimensions. This interpretation is also confirmed by the lowest value of the melting temperature observed in the calorimetric trace of the unfilled A sample (see previous section).

The inclusion of carbon nanotubes, if on the one hand decreases slightly the crystallinity degree, on the other hand, it allows preserving the crystal size, which substantially remains constant with increasing UV treatment and hence, as expected, no relevant changes are also observed in the melting temperature of the samples with increasing UV irradiation time (see Figure 9).

### 3.4. Infrared Analysis

As already reported in previous works, structural changes due to the UV treatments can be followed by studying the variations in different regions of the infrared spectrum [29,30]. In this paper, two regions have been carefully investigated; they correspond to the range of the carbonyl (C=O) stretching modes between 1700 and 1800 cm^−1^ and the range of the hydroxyl (O–H) stretching modes between 3300 and 3600 cm^−1^.

**Figure 13:**
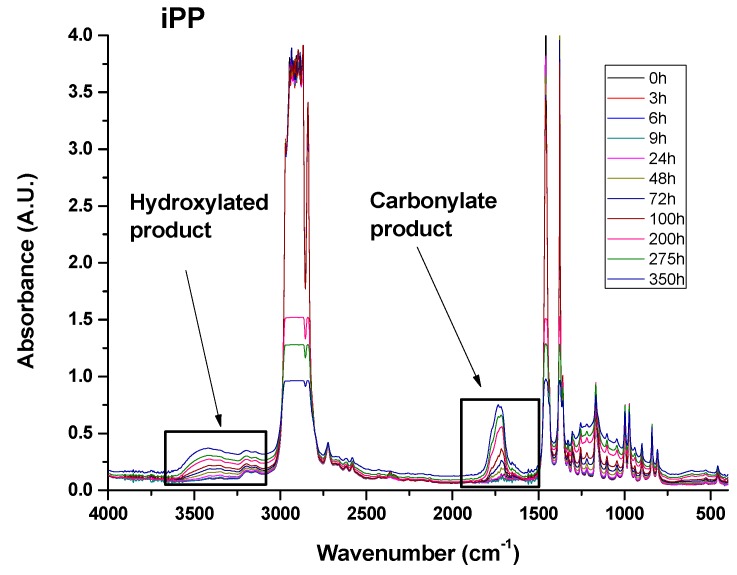
FT/IR spectra of sample A unirradiated and exposed at increasing UV irradiation times.

Figure 13 shows FT/IR spectra in the range 400–4000 cm^−1^ of sample A unirradiated and exposed at increasing UV irradiation times. The spectra of the irradiated sample show the strong broad carbonyl and hydroxyl bands as expected for unfilled samples.

**Figure 14:**
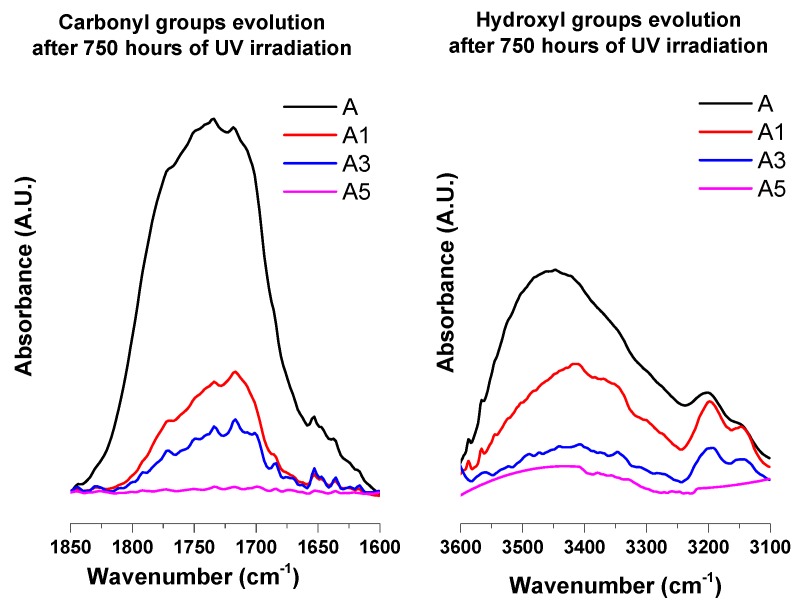
Carbonyl and Hydroxyl IR absorption bands of A, A1, A3 and A5 samples after an irradiation time of 750 h.

The enlargements of the carbonyl and hydroxyl regions of the samples A, A1, A3 and A5 after an irradiation time of 750 h are shown in Figure 14. A comparison of the different spectra highlights the strong stabilizing effect due to the inclusion of MWCNTs in the polymeric matrix. This effect seems to be strongly dependent on the amount of MWCNTs.

In fact, the spectrum of sample A5 is almost flat even after 750 h of UV treatment.

**Figure 15:**
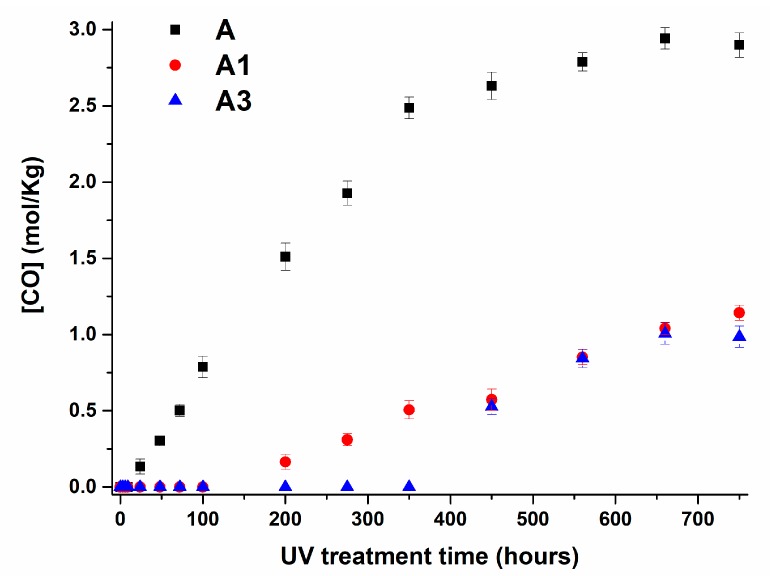
Carbonyl concentration vs. UV irradiation time for A, A1 and A3 samples.

**Figure 16:**
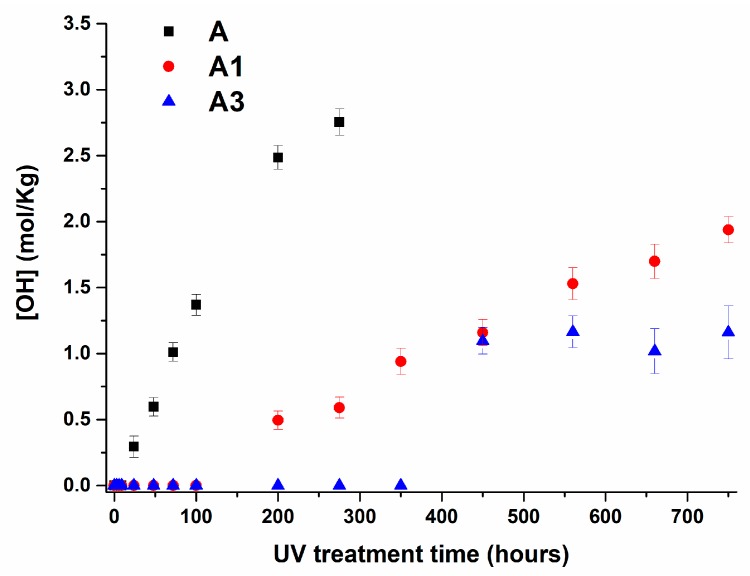
Hydroxyl concentration vs. UV irradiation time for A, A1 and A3 samples.

The evolution of carbonyl and hydroxyl groups with increasing UV irradiation times is shown in Figure 15 and Figure 16 for A, A1 and A3 samples respectively. The experimental trends for the sample A5 is not shown here as these bands are almost absent for the sample with 5 wt % of MWCNTs.

The trend of the carbonyl and hydroxyl groups highlights a very interesting effect caused by the inclusion of MWCNTs. The filler determines an induction period before the increase of the carbonyl and hydroxyl groups. The extent of this phenomenon is strictly dependent on the amount of MWCNTs. The induction period detected for sample A1 is 100 h, for sample A3, this value increase up to 350 h. Furthermore, MWCNTs determine a significant decrease in the slope of the curve in the linear region and hence lower values throughout the investigated range of time.

These experimental results on photo-oxidative behavior of polypropylene filled with MWCNTs can be discussed in the light of existing research results regarding other fillers or different polymeric matrices; for example, in light of the results obtained using carbon black (CB) as filler. CB, which is a filler extensively used in polymers for outdoor applications, has shown a dual effect on the photo-oxidative behavior of polymers. On the one hand, it protects the matrix, acting as an inner filter, decreasing the light intensity that is absorbed, thus decreasing the rate at which the polymer is oxidized. On the other hand, the absorption of light by carbon black is likely to provoke an increase of the local temperature by dissipation of the photon energy into thermal, determining an increased oxidation rate by a thermally induced process. Competition between these two antagonistic effects depends on several factors such as the percentage and the type of carbon black, the filler size, the dispersion and the structure of the polymer [29]. In the case of polypropylene, the presence of carbon black nanoparticles (20–60 nm) significantly increases UV durability of the filled polymer, especially with small particle sizes. Opposite results concerning the influence of carbon black on the photooxidation of polyethylene have shown that at low amounts, carbon black is likely to induce photodegradation [29]. A stabilizing effect was only observed for carbon black percentages higher than 5 wt %. Analogous results were reported for multi-walled carbon nanotubes in ethylene vinyl acetate (EVA) matrix [32]. For this system, the rate of photooxidation was investigated with increasing MWCNT concentrations. Increasing the amount of nanotubes increases the filter effect, enhancing the dissipation of the thermal energy, and leading to a reduction in the rate of photo-oxidation of EVA matrix. In our case similar results are obtained, although, respect to the previous cases, the stabilizing effect regarding UV exposure is highly improved, even for low nanotube concentrations (1 wt %). Indeed, after 350 h of UV exposure, the pristine sample is strongly degraded, whereas for the inclusion of 1 wt % and 3 wt % of MWCNTs in the sample, the photooxidative reactions are just at beginning of the photooxidative process (see Figure 15 and Figure 16). The nanocomposites A1 and A3 reach a degradation state at 750 h of irradiance similar to those obtained for the unfilled sample at 60 h and 100 h respectively; while an amount of 5 wt % of MWCNT makes the nanocomposite A5 still immune also at 750 h of UV treatment, confirming the exceptional property of MWCNTs in acting as UV stabilizer. The antioxidant property of MWCNTs has also highlighted by Billingham et al. for many thermoplastic matrices [63]. A relevant support on the ability of carbon nanotubes to act as antioxidant nanofiller is also provided by numerous other studies aimed at understanding the electronic conductivity of carbon nanotubes. They have shown that exposure of single-walled carbon nanotubes to air or oxygen can have a strong influence on their electronic transport properties [64,65,66,67,68,69,70]. Recent results of Rao and co-workers [71,72] suggest that UV-light excitation of O_2_ molecules into a more reactive state is a possible process responsible for the oxygen adsorption to carbon nanotubes (nanotube oxidation). They also report that O_2_ molecules are very weakly bonded (physisorbed) to perfect nanotubes while they interact very strongly with nanotube lattice defects giving rise to strong chemical bonding and significant charge transfer from nanotubes to the O_2_ molecules. They conclude that UV-light excitation of the O_2_ molecules from their ground state to a higher-energy state can give rise to a significant reduction in the activation energy for O_2_ molecule chemisorption and increases significantly the rate of nanotube oxidation. These conclusions are very useful to explain our results about the ability of carbon nanotubes to act as antioxidants even for the lowest MWCNT concentration, which is 1 wt %. O_2_ molecule bonded on the walls of carbon nanotubes that are well distributed in the polymeric matrix can locally saturate the matrix making the oxygen not available for the first stages of oxidative phenomena. The subtraction of oxygen molecules from the polymer chain oxidation extends the induction period before the trigger of photo-oxidative reactions. In fact, no degradation product containing oxygen is detectable in our spectra within the space of 200, 350 and 750 h of UV treatment for the samples with 1, 3 and 5 wt % of MWCNTs respectively.

### 3.5. Electrical Properties

The performance of carbon nanotubes in a polymer matrix is well above traditional fillers such as carbon black or ultra-fine metal powders. MWCNTs embedded in polymeric matrices are leading to many promising applications. The employment of nanocomposites based on MWCNTs may concern the use as plastics such as automobile body panels, paint, tires and as flame-retardants matrices. MWCNTs embedded in polymers have proved to have advantages also for sensing applications. With the effort from the CNT industry during the past 10 years, the price of multi-walled CNTs have strongly decreased and the productivity increased making possible the commercial and hence the industrial application of nanocomposites based on MWCNTs.

The inclusion of MWCNTs into the polymer resin significantly increases the electrical conductivity. In fact, the effect of MWCNTs on the electrical conductivity of epoxy polymers is well known in literature [73,74]. The conduction in MWCNT/composites has been explained by considering that conductive paths, causing the material to convert from an insulator to a conductor, are formed in the composite when MWCNT concentration increases over a threshold value *x*_c_. The formation of conductive network can be explained in terms of percolation theory.

Percolation theory defines an insulator-conductor transition and a corresponding threshold of the conductive filler concentration *x*_c_, via the equation:

σ = σ_0_ (*x* − *x*_c_)*^t^* (3)

where *x* is the weight fraction of MWCNTs, t an exponent depending on the system dimensionality [75,76] and σ_0_ is a scaling factor that is comparable to the effective conductivity of the filler [77].

**Figure 17:**
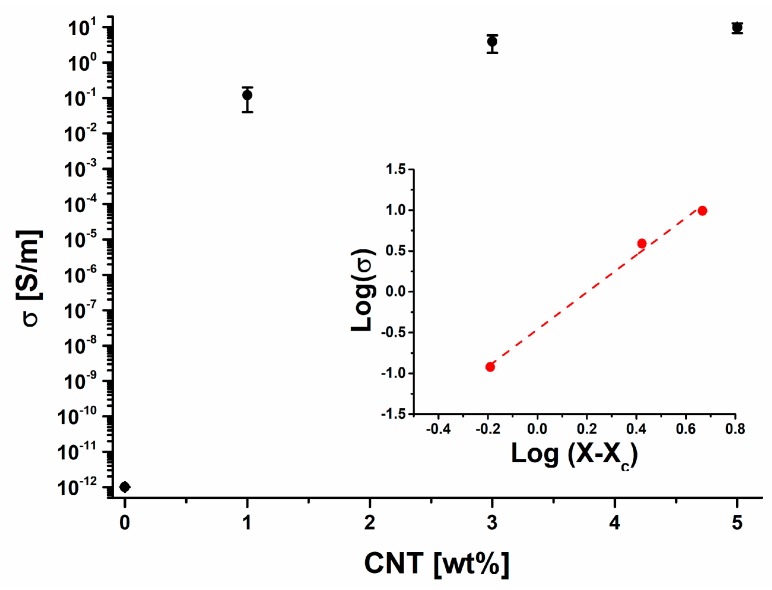
DC volume conductivity of the nanocomposites versus filler weight percentage. The inset shows the log–log plot of the electrical conductivity (σ) as a function of (*x*–*x*_c_) with the best fitted line.

As shown in Figure 17, the composite exhibits the typical abrupt increase of the conductivity predicted by the percolation theory, with the electrical percolation *x*_c_ ≤ 1 wt %. In this paper, only prelaminar results are shown. In particular, no experimental data on the electrical conductivity of the nanocomposites containing concentration of MWCNTs lower than 1wt % have been collected because of the relevant industrial interest to investigate the effect of photooxidation on the electrical properties of the nanocomposites beyond the electrical percolation threshold (EPT).

The value 0.36 wt % of the EPT (*x*_c_) has beien obtained by the best fitting of the experimental data with the Equation (3), shown in terms log–log plot (see inset in Figure 17).

From the insets of percolation curve of Figure 17, reporting the log–log plots of the experimental electrical conductivity versus filler concentration, the value for the critical exponent *t* can be obtained as the slope of the linear fit. The estimated value, *t* = 1.7, has been found to agree with universal values (*t* ≈ 2), reflecting an effective 3D organization of the percolating structure consistent with 1D type of filler [78]. The maximum electrical conductivity of iPP composites collected from literature prepared by using various fillers as a function of filler loading are in the range values around 10 S/m [79,80]. It can be observed that in case of carbon nanotube and carbon nanofibers reinforced PP, the high electrical conductivity can be achieved at relatively low filler loading 10–15 wt %. In our case, these values are achieved with a lower amount of filler (9.86 S/m for the sample filled with 5 wt % of carbon nanotubes) with respect those obtained in the literature [79,80]. The electrical conductivity of samples A3 and A5 has been evaluated also for an irradiation treatment between 0 and 200 h. As expected, no changes in the electrical conductivity of the samples have been detected in the investigated range of treatment. Further studies on this aspect dealing with the behavior of the electrical properties of the nanocomposites at lower filler concentration are now underway and will be presented in a forthcoming paper.

The low electrical percolation threshold and the high electrical conductivity value obtained for the nanocomposites, in addition to the high thermal and photoxidative stability, allow to fulfill many industrial requirements where the employment of the nanocomposites is scheduled for outdoor applications.

In particular, CNT based conductive polypropylene can provide high level of radio frequency (RF) shielding (35 dB) in electronic components and suitable performance in the protection against electrostatic discharge (ESD). Furthermore, it can be used in the automotive housing to protect inner electronic devices in vehicle wing mirrors. Samples A1, A3 and A5 are promising candidates for the mentioned applications due to their impressive photooxidative stability.

## 5. Conclusions

CNTs based conductive polypropylene can be applied in several industrial fields ranging from automotive to electronic industry. The availability of a nanocomposite strongly stable to the photooxidative degradation represents a practical and heartfelt necessity, which, if satisfied, could lead to a massive production of these nanocomposites in several industrial sectors. In this context, the effect of MWCNTs on the photooxidative behavior of nanocomposites based on the more common α polymorph of isotactic polypropylene has been studied. The results have been compared to the behaviour of the unfilled iPP sample. Thermal results highlight the role of MWCNTs in determining a protective action type barrier effect on polymer matrix. This may be due to restriction of macromolecules mobility caused by carbon nanotubes which prevents the transport of the product of photodegradation to the polymer matrix. Infrared results here shown highlight that the unfilled Polypropylene is very sensitive to ultraviolet (UV-A) photooxidation, whereas the nanocomposites show a strong stabilizing photooxidative effect of the carbon nanotubes even for the lowest MWCNTs concentration (1 wt %). Research studies aimed at determining the electronic conductivity of carbon nanotubes have shown possible chemisorption of oxygen molecules, not excluding the possibility that oxygen may also absorb on the inside of nanotubes with open ends. Such a mechanism could be responsible for abstracting oxygen molecules from the polymer chains reducing the rate of photo-oxidative reactions inside the amorphous phase in the first stage of photo-oxidative process.

The radiative treatment determines a complex structural and morphological reorganization in terms of crystallinity, crystal dimensions, and formation of groups due to the fotooxidative reactions in the unfilled sample. This rapid reorganization was not found in the UV treated nanocomposites. The formation of groups due to the fotooxidative reactions is very hindered or slowed down by the presence of nanotubes. MWCNTs are very effective in the protection of the polymeric matrix. The entity of this protection is affected by the amount of CNTs embedded in the matrix. FT/IR investigation highlights that MWCNTs determine an induction period before the increase of the carbonyl and hydroxyl groups in the polymeric matrix. The induction period detected for sample A1 is 100 h, for sample A3, this value increase up to 350 h, while for A5 sample, at 750 h of UV treatment, which is the longer period investigated in this work, the induction period is still unfinished. Data here shown highlight that MWCNTs determine a significant decrease in formation rate of Carbonyl and Hydroxyl groups also for the lowest concentration of MWCNT investigated.

## Figures and Tables

**Table 1:** Main physical properties of unfilled iPP.

Physical Property	Value
Melt index	35 g/10 min (230 °C/2.16 kg)
Mol wt	average *M*_n_ ~ 50,000
	average *M*_w_ ~ 190,000
Hardness	98 (Rockwell R, ASTM D 785-A)
Melting Temperature	*T*_m_ 160–165 °C
Density	0.9 g/mL at 25 °C (lit.)
